# Linezolid Pharmacokinetics and Its Association with Adverse Drug Reactions in Patients with Drug-Resistant Pulmonary Tuberculosis

**DOI:** 10.3390/antibiotics12040714

**Published:** 2023-04-06

**Authors:** Chandrasekaran Padmapriyadarsini, Rajesh Solanki, S. M. Jeyakumar, Anuj Bhatnagar, M. Muthuvijaylaksmi, Bharathi Jeyadeepa, Devarajulu Reddy, Prashanth Shah, Rathinam Sridhar, Vikram Vohra, Namrata Kaur Bhui

**Affiliations:** 1 ICMR-National Institute for Research in Tuberculosis, Chennai 600031, India; 2 B.J. Medical College and Hospital, Ahmedabad 380016, India; 3 Rajan Babu Institute of Pulmonary Medicine and Tuberculosis, New Delhi 110009, India; 4 Government Hospital of Thoracic Medicine, Chennai 600047, India; 5 National Institute for Tuberculosis and Respiratory Diseases, New Delhi 110030, India; 6 Grand TB Hospital, Mumbai 400015, India

**Keywords:** linezolid, pharmacokinetics, myelosuppression, neuropathy, therapeutic drug monitoring

## Abstract

We evaluated the relationship between the pharmacokinetic parameters of linezolid (LZD) and development of adverse drug reactions (ADRs) in patients with pulmonary drug-resistant tuberculosis. A prospective cohort of adults with pulmonary multidrug-resistant tuberculosis with additional resistance to fluoroquinolone (MDR-TB_FQ+_) received treatment with bedaquiline, delamanid, clofazimine, and LZD. Blood samples were collected during weeks 8 and 16 at eight time points over 24 h. The pharmacokinetic parameters of LZD were measured using high-performance liquid chromatography and associated with ADRs. Of the 165 MDR-TB_FQ+_ patients on treatment, 78 patients developed LZD-associated anemia and 69 developed peripheral neuropathy. Twenty-three patients underwent intense pharmacokinetic testing. Plasma median trough concentration was 2.08 µg/mL and 3.41 µg/mL, (normal <2 µg/mL) and AUC_0-24_ was 184.5 µg/h/mL and 240.5 µg/h/mL at weeks 8 and 16, respectively, showing a linear relationship between duration of intake and plasma levels. Nineteen patients showed LZD-associated ADRs-nine at week 8, twelve at week 16, and two at both weeks 8 and 16. Thirteen of the nineteen had high plasma trough and peak concentrations of LZD. A strong association between LZD-associated ADRs and plasma LZD levels was noted. Trough concentration alone or combinations of trough with peak levels are potential targets for therapeutic drug monitoring.

## 1. Introduction

Drug-resistant tuberculosis (DR-TB) remains a public health threat, threatening the achievement of End TB targets [1] due to an increase in estimated burden, difficulty in management, and an increased mortality from the disease [1]. The poor treatment outcome of DR-TB underscores the need for more effective, shorter, and patient-friendly therapies with a combination of new and repurposed drugs [2]. One such repurposed drug that has been found to be efficacious in the DR-TB treatment regimen is linezolid (LZD), an oxazolidinone class antibiotic [3,4]. The World Health Organization regrouped drugs used in the management of DR-TB in 2019, with LZD, bedaquiline (BDQ), and levofloxacin/moxifloxacin being upgraded to “group A” drugs that must be present in DR-TB regimens alongside group B drugs [5]. However, the adverse events associated with LZD are many, with the most common being myelosuppression, peripheral neuropathy, and optic neuropathy, which result in permanent discontinuation of the drug [6,7,8].

Adverse events with LZD are secondary to mitochondrial toxicity, which appears to be dose- and duration-dependent, due to its narrow therapeutic index and uncertainty around optimal dosing [9]. It has been suggested that high trough and cumulative concentrations of LZD in the blood can lead to adverse drug reactions (ADRs) [10,11]. Some of the ADRs, if found early and treated by LZD dose reduction or discontinuation, are reversible, while others result in irreversible damage [12,13]. However, LZD is still being used in the programmatic management of DR-TB in many countries. We hypothesized that estimating plasma LZD levels and correlating them with clinical conditions may help in the early identification of adverse events. This may prompt the treating physician to lower the drug dose without jeopardizing its efficacy, thereby avoiding permanent damage or disability caused by LZD toxicity. We, therefore, estimated the plasma level of LZD and associated it with the adverse events in a group of pre-extensively drug-resistant pulmonary TB patients who were being treated with a LZD-based regimen.

## 2. Results and Discussions

### 2.1. Participants Profile

A total of 287 MDR-TB_FQ+_ patients were screened, of whom 165 eligible patients were started on a regimen containing LZD, BDQ, DLM, and CFZ. The average age and weight were 27 years and 48.0 kg, respectively (Table 1). Before starting treatment, the median hemoglobin was 11.4 mg/dL (range: 8.1 to 16.5). During treatment, 78 patients developed anemia of varying severity after 8 weeks of treatment while 69 developed signs of peripheral neuropathy after 16 weeks of treatment. The participants in good general condition and without any drug interruptions or active drug-related adverse events before the time of the PK study were sequentially approached to participate in the PK study. Those willing to have blood drawn at eight time points were recruited for an intensive PK study (22 patients), while those willing to participate in the PK study but were hesitant to have so many blood draws were recruited to a sparse PK study with blood drawn at four time points only.

### 2.2. Pharmacokinetic Parameters of Linezolid

**Figure 1:**
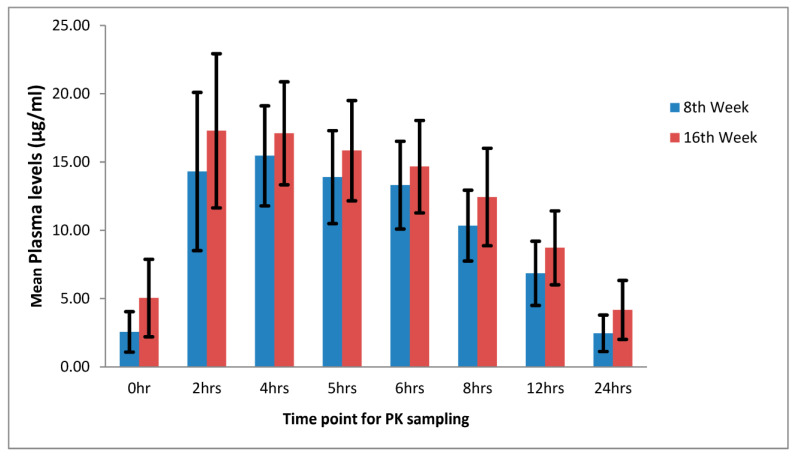
Mean plasma concentration of linezolid (with 95% CI) at different time points in study participants on a linezolid-containing regimen for treatment of drug-resistant tuberculosis.

Twenty-three participants consented and underwent intense PK testing (22 at week 8 and 19 at week 16 after a steady state of LZD was attained. One participant did not undergo PK testing at week 8 but participated at week 16). Eighteen participants underwent the testing at both weeks 8 and 16. The majority (78%) were male, with a mean age of 30 years (range: 18–55 years) and a mean BMI of 20.1 kg/m^2^. Tablet LZD was started at a dose of 600 mg daily in all participants, and the median duration of LZD therapy, excluding treatment interruptions, was 168 days. The median C_max_ was 18.3 μg/mL and 18.9 μg/mL while the median C_min_ was 2.08 µg/mL and 3.41 µg/mL at weeks 8 and 16, respectively (Table 1). The median AUC_0-24_ was 184.5 µg/h/mL and 240.5 µg/h/mL at weeks 8 and 16, respectively (Figure 1). Similarly, C_max_ > 20 µg/mL is considered supratherapeutic. Of the 23 participants, 8 participants each at weeks 8 and 16 had supratherapeutic levels with two of them showing high levels at both 8 and 16 weeks (Table 1). There was also a difference in clearance at week 16 when compared with week 8 as observed in the non-compartmental PK analysis. The plasma concentration and AUC increased with longer exposure to LZD showing a linear relationship between the duration of intake and plasma level (Figure 1).

### 2.3. Relationship between LZD Exposure and Toxicity

Table 2 shows the relationship between LZD trough concentration (C_min_), therapeutic levels with ADRs, and their outcomes. Of the 23 patients who underwent PK testing, twenty-one ADRs were observed in 19 patients—nine around week 8, twelve around week 16, and two at both weeks 8 and 16. Of them, 13 had plasma LZD levels above therapeutic levels (C_max_ > 20µg/mL) while 6 had normal therapeutic levels of LZD (Table 2). Eighteen patients had high trough concentrations (C_min_ > 2μg/mL)—three at week 8, seven at week 16, and eight at both 8 and 16 weeks (Table 2). Of the 19 participants with ADR, 13 patients had high trough concentrations as well as supratherapeutic levels of LZD. The most frequently encountered ADRs in this cohort are listed below. 

#### 2.3.1. Anemia and Plasma Levels of LZD

Among the nineteen patients with ADR, ten patients developed anemia of varying grades (Table 2)—three at week 8, five at week 16, and two at both weeks 8 and 16. As the majority of them were grades I and II, LZD was continued with hematinic support. In four patients (grade III and IV anemia at week 16), LZD was withheld temporarily and later reintroduced at a lower dose of 300 mg while one patient also received a blood transfusion. All eleven patients completed treatment and were declared cured. Subsequently, when PK parameters were correlated, eight patients had high trough as well as therapeutic levels while two patients had a high trough concentration alone. One patient who had a high trough concentration at both weeks 8 and 16 and high therapeutic levels at week 16, later developed grade III anemia at week 18, which was not picked up earlier.

#### 2.3.2. Peripheral Neuropathy (PN)

Peripheral neuropathy was observed in six patients (three at week 8 and three at week 16). No drug interruption was done as four were of grade I severity and two were of grade II severity that were managed symptomatically. Five patients completed treatment and were declared cured; one patient died at week 20 of treatment due to disease severity. Subsequent PK correlation revealed high trough as well as therapeutic levels in four patients while two patients had a high trough concentration alone.

#### 2.3.3. Blurring of Vision and Optic Neuritis

Blurring of vision was observed in two patients—one at week 5 and another at week 12. The ophthalmologist ruled out optic neuropathy in the first patient and hence LZD was continued, treatment was completed, and the patient was declared cured. The second patient at week 12 was diagnosed with early signs of optic neuropathy and was advised to discontinue LZD. Subsequent PK correlation showed a high trough concentration alone in the first patient while the second patient with early changes of optic neuropathy had both high trough concentration as well as therapeutic levels.

The participants in the study received bedaquiline, delamanid, and clofazamine along with linezolid. The major ADRs reported were hyperpigmentation secondary to clofazamine use, followed by anemia and peripheral neuropathy, attributable to linezolid. None of the patients developed QTc prolongation more than 500 msec, a common AE of bedaquiline [14]. No potential interaction was noticed between the other drugs used in the regimen and the development of ADRs.

### 2.4. Sparse PK

Sparse PK was conducted in 32 patients (25 patients in week 8 and 24 patients in week 8, with 17 patients coming in both weeks), independent of intense PK. Of the 32 patients, thirty-two ADRs were observed in 22 patients—ten at week 8, five at week 16, and seven at both weeks 8 and 16 (Table 2). Ten patients did not have any adverse events.

To summarize, no statistically significant correlation was observed between the occurrence of ADRs and the trough concentration of LZD. However, significance was observed between anemia and therapeutic levels of LZD when both intense and sparse PK patients were combined (*p* value = 0.03) (Table 3). There was no correlation between therapeutic levels and the occurrence of peripheral neuropathy or blurring of vision.

### 2.5. Discussions

In our cohort, anemia and peripheral neuropathy were encountered frequently but were all successfully managed in the clinical settings. We noticed a high trough and peak concentration among patients developing ADRs. In a South African cohort of DR-TB patients, a LZD trough concentration of ≥2.5 mg/L was associated with nearly 3-fold increased odds of anemia and thrombocytopenia but not neuropathy [15]. In another cohort of Korean XDR-TB patients, a high proportion of clinical events were observed when the trough threshold reached >2 mg/L [10]. All patients with a mean LZD trough concentration >2 mg/L developed clinical toxicity, whereas only 50% of those with LZD trough <2 mg/L developed clinical toxicity. Besides these cohort studies, LZD trough concentrations has also been shown to be associated with hematological toxicity in other studies including a mouse model [16,17]. These suggest a role for LZD trough concentration as a potential target for therapeutic drug monitoring to reduce adverse events. Even in our cohort, 13 of 19 patients with ADRs had a trough concentration >2 mg/L and all patients with a LZD trough concentration >2 mg/L along with C_max_ above the therapeutic range manifested ADRs.

According to studies, patients with an AUC_0-24_ more than 120.69 mg/L h may experience low hemoglobin 1–7 days after the end of LZD treatment, and those with an AUC_0-24_ greater than 92.88 µg/h/mL may experience thrombocytopenia 8–15 days after the end of LZD treatment [18]. However, we did not find any association between AUC level and the development of ADRs. In addition, our cohort demonstrated that as LZD therapy continued, the cumulative response increased. An increase in the C_min_ and AUC_0-24_ values at week 16 relative to week 8 suggests an exposure–response association with LZD.

The substantial interindividual variability in the PK of LZD results in an unacceptably high proportion of patients with either inadequate or potentially toxic concentrations after the administration of a fixed dose of 600 mg, particularly when the drug is administered for an extended period [19]. Linezolid has a narrow therapeutic window and is known for its severe and irreversible ADRs. According to the product insert, its use should be limited to 28 days; however, for MDR/XDR-TB, LZD is used for 6–12 months or even longer. Therapeutic drug monitoring has a critical role in detecting drug exposure and optimizing the dose. This is the first study to examine the relationship between LZD blood levels and the development of ADRs in a nation with a high burden of both TB and malnutrition. We have demonstrated that a larger trough level was reached when the daily dosage of LZD is 600 mg. There was also a difference in clearance of LZD, from the 8th to 16th week, and this could be due to alterations in renal function with the continued use of LZD, as noticed by other investigators [20,21].

During therapy, it was also discovered that individuals with high trough levels experienced one or more ADRs. As all study participants received a combination of BDQ and DLM for the first time along with LZD, they were all closely monitored, allowing us to identify and swiftly treat all ADRs. In our cohort, LZD dose was reduced from 600 mg to 300 mg in patients who developed ADRs. These patients not only showed resolution of the ADRs but also had favorable outcome until the end of study. Unfortunately, a repeat drug level estimation while on a reduced dose of LZD was not performed and hence, we are not in a position to comment on the feasibility of lowering the dose without it resulting in sub-therapeutic concentrations.

There are limitations to consider when interpreting our findings. C_max_ showed a significant correlation with the development of ADRs; however, given the inter-individual heterogeneity in T_max_, it is difficult to recommend a fixed time point for therapeutic drug monitoring. Due to the small sample size of the sub-study, predictors for the incidence of adverse events (age, gender, weight, disease status, smear status, co-morbidities) could not be assessed in association with the PK parameters like AUC which may need a full intense PK in larger numbers. Additionally, because PK testing was not performed in real-time, but rather in batches and delayed, we were unable to alter the LZD dosage in a timely manner. However, a strength of our study is that it accurately represents real-world circumstances—participants’ unwillingness to undergo frequent blood draws and the late presentation of ADRs. Additionally, in real-world program settings, parameters for a single blood draw that can be used as a surrogate to identify ADRs early and reduce the LZD dose will be better accepted than an intense PK and our study has shown that trough level can be used as such a marker.

## 3. Materials and Methods

### 3.1. Study Design and Setting

A sub-study in the BEAT-India study, described elsewhere in detail, was undertaken to understand the pharmacokinetic–toxicity relationship of LZD. In brief, the BEAT-India study enrolled adults with pulmonary DR-TB with additional resistance to fluoroquinolones (MDR-TB_FQ+_) and/or second line injectable (MDR-TB_SLI+_) between 2019 and 2021 at five sites in India and were initiated on an all-oral short-course regimen consisting of BDQ and LZD along with delamanid (DLM), and clofazimine (CFZ) for 6–9 months [14]. All participants received 600 mg of LZD daily along with the standard dose of BDQ, DLM, and CFZ and were followed at periodic intervals, as per the study protocol, throughout the treatment period to assess the efficacy and safety of the study regimen. To monitor adverse events, at follow-up visits, a detailed clinical and systemic examination was done along with blood tests, including a complete blood count, and liver and renal function tests. A limited number of neurologic and ophthalmic examinations were done by the site physician to assess for drug-induced toxicity. In the case of any abnormality, the patient was immediately sent to a neurologist or an ophthalmologist (as appropriate) for a detailed and thorough examination and management. The neurologic exam ruled out motor and sensory deficits, paresthesia, tendon reflexes, and cranial nerve abnormalities, while the ophthalmologic exam included tests for visual acuity loss, visual color abnormalities, and scotomas. If required, nerve conduction velocity (NCV) testing was performed to assess motor and sensory injuries and confirm the diagnosis.

### 3.2. Intense and Sparse Pharmacokinetics

An intense pharmacokinetic (PK) study was conducted in a subset of volunteers at weeks 8 and 16 of treatment. In the event of drug-induced adverse events, the drug was temporarily withheld and the dose was reduced. On the day of intensive PK, it was ensured that the participant had been on all four drugs for at least one week without interruption. Blood samples were collected at eight time points over 24 h: 0, 2, 4, 5, 6, 8, 12, and 24 h. The 0 h sample was taken on the day of PK before the intake of ATT, and all other sample collections were performed at specified hours after drug intake at week 8 (Day 57). The same series of blood collection was repeated at week 16 (Day 113). A PK study was not done if the participant had other drug-related adverse events (due to BDQ, DLM, or CFZ) during treatment or if they had missed doses during the week before the planned PK study.

We also tried to evaluate if a sparse PK sampling at fewer time points could give similar results as an intense PK, as this would be more cumbersome for patients. As a result, a sparse PK sampling was performed in another subset of willing volunteers (32 patients), who accepted to take part in the PK study, but were not willing to stay back in the hospital for an extended period of time and were reluctant for a blood draw at so many time points between 8 and 16 weeks of treatment. The blood samples were collected at four time points at 3, 4, 5, and 6 h post-drug intake and checked for associations of a few PK parameters with the occurrence of ADRs.

### 3.3. Drug Estimation and Analysis

As previously described, plasma LZD concentrations were determined using a reverse-phase high-performance liquid chromatography (HPLC) technique [22]. Proteins were precipitated by combining 100 μL of plasma sample with 200 μL of acetonitrile, mixing vigorously, and centrifuging at 7000 rpm for 10 min. The supernatant (200 μL) was transferred to a new tube, evaporated under dry nitrogen, and reconstituted with a 100 μL mobile phase. Then, 20 μL of this mixture was injected into the HPLC machine for analysis. For samples whose concentration exceeded the range of 0.2–20 g/mL, a suitable dilution was performed.

As Cmax was achieved at 4 h of drug intake, this was estimated for both sparse and intense PK and used for analysis. As the ‘0’ hour blood sample collection was not performed for the sparse PK, we could not calculate Cmin and AUC for sparse PK and hence these parameters could not be combined and we used only intense PK data for these analyses.

### 3.4. Statistical Analysis

Based on the plasma concentration of LZD at different time points, certain PK parameters, namely C_max_, T_max_, AUC, half-life, and clearance, were calculated. A non-compartmental model was applied for the LZD PK estimations, and data analysis was performed using SPSS software (IBM version 25.0, IBM Corp., Armonk, NY, USA)**.** The data were presented as descriptive statistics using frequencies and percentages. A non-parametric test (Mann–Whitney test) was used to evaluate the significance of the difference between the groups at the two time points. The therapeutic range of LZD is 10–20 µg/mL and a trough concentration (C_min_) <2 μg/mL is considered normal [23]. The Division of AIDS (DAIDS) Table for Grading the Severity of Adult 2 and Pediatric Adverse Events [24] was used to classify the severity of neurologic and ophthalmologic adverse effects [24]. Given the small sample size, we could not perform any regression-based or adjustment for covariates analysis.

## 4. Conclusions

As we progress towards precision medicine, individualization of LZD doses should be addressed to maximize efficacy and decrease the toxicity associated with LZD. The association between blood levels of LZD and the occurrence of ADRs has emphasized the necessity for therapeutic drug monitoring in DR-TB patients receiving a LZD-containing regimen. As a viable target for TDM, a trough concentration alone or a combination of trough and peak levels requires further evaluation.

## Figures and Tables

**Table 1:** Demographic and pharmacokinetic parameters of participants who underwent intense pharmacokinetic testing for tablet linezolid.

Characteristics		Week 8 [n = 22]	Week 16 [n = 19]
Weight in kg [mean (SD)] Min–Max		54.9 (12.7)	54.4 (9.8)
		(36.0–92.0)	(36.0–71.1)
Body mass index	Mean (SD)	20.1 (4.9)	20.4 (4.1)
Min–Max		(14.2–32.1)	(14.4–29.3)
C_max_ (μg/mL)	Median	18.3 (15.4–22.3)	18.9 (16.1–21.9)
C_min_ (μg/mL)	Median	2.08 (1.25–2.90)	3.41 (2.26–5.07)
AUC_0–24_ (ug/h/mL)	Median (IQR)	184.5 (166.2–223.1)	240.5 (191.6–275.7)
Tmax (h)	Median (IQR)	2 (2.0–4.0)	2 (2.0–4.0)
Clearance (L)	Median (IQR)	2.9 (2.4–3.3)	2.1 (1.7–2.8)
Half Life (Hrs)	Median (IQR)	6.9 (5.8–7.9)	8.8 (7.4–11.3)
**Therapeutic Range of LZD (10–20 µg/mL) n (%)**			
Sub Therapeutic (<10)		1 (4.5%)	0
Therapeutic (10–20)		13 (59.1%)	11 (57.9%)
Supra Therapeutic (>20)		8 (36.4%)	8 (42.1%)

SD = standard deviation; IQR = interquartile range; C_max_ = maximum concentration; C_min_ = minimum concentration; AUC_0-24_ = area under the time–concentration curve; T_max_ = time to maximum concentration.

**Table 2:** Co-relation between the pharmacokinetic values of linezolid and adverse events with their outcome among study participants on a linezolid-containing regimen for drug-resistant tuberculosis.

Case No	Linezolid Pharmacokinetics				Therapeutic Range		Adverse Events		Outcome at End of Treatment
	C_max_ 8th Week	C_max_ 16th Week	C_min_ 8th Week	C_min_ 16th Week	C_max_ >20 μg/mL	C_min_ >2 μg/mL	At 8th Week	At 16th Week	
1	PK Not done	>20	PK Not done	>2	YES	YES	-	Grade I Peripheral Neuropathy	Death at 20 W
2	10 to 20	10 to 20	<2	>2	NO	YES	-	-	Cured
3	>20	PK Not done	>2	PK Not done	YES	YES	-	Optic Neuritis at 12 W	Withdrawn due to ADR
4	>20	10 to 20	>2	<2	YES	YES	-	Grade I Anemia	Cured
5	10 to 20	>20	>2	>2	YES	YES	-	Grade I Anemia	Cured
6	10 to 20	10 to 20	<2	<2	NO	NO	-	-	Cured
7	10 to 20	>20	<2	>2	YES	YES	Grade II Peripheral Neuropathy	-	Cured
8	>20	>20	>2	>2	YES	YES	-	Grade III Anemia	Cured
9	>20	>20	>2	>2	YES	YES	Grade I Peripheral Neuropathy	-	Cured
10	10 to 20	10 to 20	<2	<2	NO	NO	Grade I Anemia	-	Cured
11	10 to 20	>20	<2	>2	YES	YES	-	-	Cured
12	10 to 20	PK Not done	>2	PK Not done	NO	YES	-	Grade III Anemia	Cured
13	10 to 20	10 to 20	>2	>2	NO	YES	-	Grade II Peripheral Neuropathy at 22 W	Cured
14	10 to 20	10 to 20	<2	>2	NO	YES	Grade II Anemia at 1 W	-	Cured
15	10 to 20	10 to 20	<2	>2	NO	YES	Blurring of vision at 5 W	-	Cured
16	10 to 20 *	>20	>2 *	>2	YES	YES	-	-	Cured
17	>20 **	10 to 20	>2 **	>2	YES	YES	-	Grade III Anemia	Cured
18	<10	10 to 20	<2	>2	NO	YES	-	-	Cured
19	>20	10 to 20	>2	>2	YES	YES	Grade I Anemia at 1 W	-	Cured
20	10 to 20	>20	<2	>2	YES	YES	Grade I Anemia at 2 W	Grade I Anemia	Cured
21	>20	10 to 20	>2	>2	YES	YES	-	Grade I Peripheral Neuropathy	Cured
22	>20 **	PK Not done	>2 **	PK Not done	YES	YES	Grade III Anemia at 2 W	Grade IV Anemia 14 W	Cured
23	10 to 20	PK Not done	>2	PK Not done	NO	YES	Gd I Peripheral. Neuropathy at 3 W	-	Cured

Note: * developed grade III anemia at week 18; ** dose interruption and reintroduction.

**Table 3:** Occurrence of adverse events and estimated pharmacokinetic parameters in the steady state (Cmin, Cmax, and AUC) among the study participants on a linezolid-containing regimen for drug-resistant tuberculosis.

Adverse Event	Intense Pharmacokinetic Sampling						Combining Intense and Sparse Pharmacokinetic Results		
	C_min_ (μg/mL)			AUC_0–24_ (μg/mL)			C_max_ (μg/h/mL)		
	No	Yes	*p*-Value	No	Yes	*p*-Value	No	Yes	*p*-Value
Peripheral Neuropathy	19	4	0.54	19	4	0.74	38	17	0.89
	3.9 ± 2.3	3.2 ± 1.1		229.8 ± 76.5	216.3 ± 42.2		21.4 ± 6.1	21.2 ± 4.3	
Anemia	15	8	0.24	15	8	0.78	23	32	0.029
	3.3 ± 1.6	4.7 ± 2.8		223.6 ± 52.7	234.7 ± 101.1		19.5 ± 4.5	22.6 ± 5.9	
Blurring of vision	21	2	0.94	21	2	0.63	53	2	0.96
	3.8 ± 2.2	3.9 ± 1.4		225.2 ± 72.7	251.5 ± 64.4		21.3 ± 5.6	21.5 ± 4.9	

C_max_ = maximum concentration; C_min_ = minimum concentration; AUC_0-24_ = area under the time–concentration curve.

## Data Availability

Since the manuscript involves patient data, in view of patient confidentiality, access to deidentified data is available on request to the Principal Investigator.
